# Association between anion gap and mortality of aortic aneurysm in intensive care unit after open surgery

**DOI:** 10.1186/s12872-021-02263-4

**Published:** 2021-09-23

**Authors:** Yijing Gao, Zilin Hong, Runnan Shen, Shiran Zhang, Guochang You, Jie Chen, Xushun Guo, Senyi Peng, Kai Huang

**Affiliations:** 1 Department of Cardiovascular Surgery, Sun Yat-Sen Memorial Hospital, Sun Yat-Sen University, No. 33, Yingfeng Road, Haizhu District, Guangzhou, 510000 Guangdong Province China; 2 Zhongshan School of Medicine, Sun Yat-Sen University, No. 58, Zhongshan Rd. 2, Guangzhou, 510080 Guangdong Province China; 3 Department of Cardiovascular Surgery, Guangzhou Red Cross Hospital, No. 396, Tongfu Middle Road, Guangzhou, 510220 Guangdong Province China

**Keywords:** Aortic aneurysm, Anion gap, Prognosis, Open surgery, Intensive care unit

## Abstract

**Background:**

There has not been a well-accepted prognostic model to predict the mortality of aortic aneurysm patients in intensive care unit after open surgery repair. Otherwise, our previous study found that anion gap was a prognosis factor for aortic aneurysm patients. Therefore, we wanted to investigate the relationship between anion gap and mortality of aortic aneurysm patients in intensive care unit after open surgery repair.

**Methods:**

From Medical Information Mart for Intensive Care III, data of aortic aneurysm patients in intensive care unit after open surgery were enrolled. The primary clinical outcome was defined as death in intensive care unit. Univariate analysis was conducted to compare the baseline data in different groups stratified by clinical outcome or by anion gap level. Restricted cubic spline was drawn to find out the association between anion gap level and mortality. Subgroup analysis was then conducted to show the association in different level and was presented as frost plot. Multivariate regression models were built based on anion gap and were adjusted by admission information, severity score, complication, operation and laboratory indicators. Receiver operating characteristic curves were drawn to compare the prognosis ability of anion gap and simplified acute physiology score II. Decision curve analysis was finally conducted to indicate the net benefit of the models.

**Results:**

A total of 405 aortic aneurysm patients were enrolled in this study and the in-intensive-care-unit (in-ICU) mortality was 6.9%. Univariate analysis showed that elevated anion gap was associated with high mortality (*P* value < 0.001), and restricted cubic spline analysis showed the positive correlation between anion gap and mortality. Receiver operating characteristic curve showed that the mortality predictive ability of anion gap approached that of simplified acute physiology score II and even performed better in predicting in-hospital mortality (*P* value < 0.05). Moreover, models based on anion gap showed that 1 mEq/L increase of anion gap improved up to 42.3% (95% confidence interval 28.5–59.8%) risk of death.

**Conclusions:**

The level of serum anion gap was an important prognosis factor for aortic aneurysm mortality in intensive care unit after open surgery.

**Supplementary Information:**

The online version contains supplementary material available at 10.1186/s12872-021-02263-4.

## Background

Aneurysm is the second most frequent disease of the aorta after atherosclerosis [1]. Aortic aneurysm (AA) accounts for over 10,000 deaths in America annually [2]. AA is subdivided into thoracic aortic aneurysm (TAA) and abdominal aortic aneurysm (AAA). Despite diagnostic advances, in-time diagnosis of AAs in an early stage is difficult due to lack of classic symptoms. AAAs are found in up to 8% of men aged 65 years, yet usually remain asymptomatic until they rupture [3]. Early TAAs remain asymptomatic as well, until aortic dissection or rupture occurs, which is related to a high mortality of approximately 50% [4]. With the aging of population, the incidence of aortic diseases will increase further [5].

Nowadays, surgery is still the main treatment of AA, of which open surgery repair (OSR) and endovascular aortic repair (EVAR) are the most common two. In the recent years, the latter is used more often because of its low mortality and probability of comorbidities in perioperative phase, since EVAR avoids ischemia of organ and large trauma, compared with OSR [1]. However, OSR is still an important choice of AA patients due to its efficacy within a wide range. First of all, OSR shows long-term advantages, compared with EVAR. The Dutch Randomized Endovascular Aneurysm Management (DREAM) trial shows that although EVAR is better than OSR in aspect of 30-day mortality, comorbidity and length of stay (LOS) in hospital, there is no significant difference in 6-year follow-up after surgery, and reintervention rate of EVAR is even higher than that of OSR [6]. What’s more, EVAR1, a clinical trial reports that patients who have received EVAR have a lower survival rate than patients that have received OSR in 15-year follow-up, mainly owing to an increase of secondary aneurysmal sac rupture in EVAR group [6]. Therefore, OSR is still a treatment worthy of consideration. Moreover, OSR is still the standard when dealing with some types of AA. In cases of Marfan disease and other connective tissue diseases, when dealing with TAA in the descending part of the aorta, OSR should be preferred over thoracic endovascular aortic repair (TEVAR), since there is no evidence supporting any use of TEVAR in patients with connective tissue disease, except in emergency situations in order to get initial stabilization as a bridge to definitive surgical therapy [1]. As for AAA, in patients with complex aortic anatomy, including those with aneurysms in close proximity to or involving the renal arteries, EVAR is unsuitable, and OSR remains the standard [1]. In addition, OSR is a remedy when EVAR fails or causes comorbidities. For instance, OSR is a selection of prompt treatment to secondary endoleak following EVAR and TEVAR [7]. Since OSR has high efficacy but still with problems in safety simultaneously, the prognosis of it is still worthy of enough consideration. However, it seems that the existing predictive models for AA mortality have several shortcomings, and are not useful and practical for clinical decision making [8]. Thus, new predictors of AA prognosis should be discovered. What’s more, elevated anion gap (AG) is a risk factor for mortality of critically ill patients. Kim et al indicates that corrected AG at ICU admission may be used to predict mortality in children, regardless of underlying etiology [9]. Since there had not been a research that study the relationship between AG and mortality of AA, in our previous study, we conducted a research aiming to confirm AG as a prognosis factor for prognosis of AA patients in ICU, using the patient information obtained from Medical Information Mart for Intensive Care III (MIMIC-III), and found admission serum anion gap might serve as a strong predictor of ICU mortality for AA patients [10]. However, in that study we did not include patients with thoracoabdominal aneurysm, and we did not focus on OSR specially, which has higher perioperative mortality. Since OSR has certain risk of adverse prognosis, especially in perioperative phase, it’s necessary to build and find reliable prognosis factors of OSR. By conducting the current research, we aimed to find out that whether AG had a better prognosis effect when dealing with the subgroup receiving OSR. Moreover, we wanted to use more kinds of statistical tools to find out the association between AG and clinical outcome and estimate the clinical net benefit of the predictive models.

## Methods

### Data retrieval

The data used in this study were all obtained from MIMIC-III, a free database that integrates in-hospital data of over 50,000 ICU patients in Beth Israel Deaconess Medical Center in Boston, Massachusetts from 2001 to 2012 [11]. In MIMIC-III, the diagnoses of a patient are defined by International Classification of Diseases, Clinical Modification (ICD-9-CM) [11]. Through International Classification of Diseases 9 (ICD-9) code, first hospital admission and first ICU admission of AA patients were included. Exclusion standards were as follows: (1) not first hospital and ICU admission; (2) age < 18; (3) ICU stay < 24 h; (4) without an AG record in first day after admission; (5) did not receive open surgery.

The baseline data of patients that we obtained included general condition, comorbidity, laboratory indicators on admission, treatment and severity scores. Comorbidity were defined by ICD-9 code as well on same hospital admission. Among laboratory indicators, AG, creatinine, blood urea nitrogen, partial thromboplastin time (PTT), international normalized ratio (INR), prothrombin time (PT) and white blood cell count were maximum value detected in first day of ICU admission, while bicarbonate, hematocrit, hemoglobin and platelet count were the minimum value. For missing value, we performed single imputation for the whole dataset based on the complete conditional specification and used predictive mean matching method to full-fill them [12]. A sensitivity analysis was also conducted to access the difference between original and imputed data. For those who are interested in the details of this sensitivity analysis, check Additional file 1 for more information. The data obtained from MIMIC-III were stored in PostgreSQL (Version: 10.12). Creation of materialized view and extraction of relevant data were conducted in PostgreSQL (Version: 10.12) through mimic-code for MIMIC-III [13, 14].

### Clinical outcome

The primary outcome of patients was death in ICU. The secondary outcome included death in hospital, death within 90 days and death within 365 days.

### Data analysis

Baseline data of AA patients in ICU after OSR were grouped by clinical outcomes. The continuous data’s normality was tested through Shapiro–Wilk normality test. And in two independent groups comparison, continuous variables with normal distribution would be represented by mean with standard deviation (SD), compared through t-test. Continuous variables with abnormal distribution would be represented by median and interquartile range, compared through Wilcoxon rank sum test. Categorical variables would be represented by frequency and percentage, compared through chi-square test. To find out whether AG had a predictive effect of mortality, we stratified the baseline data by AG level (< 12 mEq/L, 12–16 mEq/L, > 16 mEq/L) on ICU admission. The continuous data’s normality was tested with Shapiro–Wilk normality test. In more than two groups comparison, continuous variables with normal distribution would be represented by mean with SD, compared through one-way ANOVA test. Continuous variables with abnormal distribution would be represented by median and interquartile range, compared through Kruskal–Wallis rank sum test. Categorical variables would be represented by frequency and percentage, compared through chi-square test. To illuminate the effects of AG on risk of ICU mortality, we drew 4 restricted cubic splines (RCSs) which were adjusted for different kinds of variables or non-adjusted. The first one model was crude and not adjusted; The second model was adjusted for admission information and severity score, including admission type, age, gender, aortic rupture, Sequential Organ Failure Assessment (SOFA), Simplified Acute Physiology Score II (SAPSII) and Glasgow Coma Scale (GCS); The third model was adjusted for complication and operation, including sepsis, chronic pulmonary diseases, peripheral vascular diseases, hypertension, renal failure, coagulopathy, fluid and electrolyte disorders, extracorporeal circulation, bypass surgery, ventilation on first day and urine output on first day; The fourth model was adjusted for laboratory indicators, including bicarbonate, creatinine, blood urea nitrogen, hematocrit, hemoglobin, PTT, PT, INR, white blood cell count and platelet count.

Logistic regression was conducted to show subgroup analysis of association between serum AG and ICU mortality and was adjusted for SOFA score. Different groups were stratified by type of admission, rupture, age, gender, sepsis, chronic pulmonary diseases, renal failure, coagulopathy diseases and fluid electrolyte disorders. Finally, the result of the subgroup analysis was presented as frost plot.

We conducted some analyses to compare the risk discrimination of AG and SAPSII in aspect of different clinical outcomes, including ICU-mortality, hospital-mortality, 90-day mortality, and 1-year mortality. Area under curve (AUC) of continuous AG and SAPSII were calculated respectively and compared by Delong’s test of their correlated receiver operating characteristic (ROC) curves [15]. Net reclassification index (NRI) and integrated discrimination improvement (IDI) between AG model and SAPSII model were also calculated and tested [16].

To assess the effects of AG on ICU mortality, models including AG were analyzed through multivariable logistic regression. Among these models, one was a crude model which only includes AG gap, while the other 4 models were adjusted for different variable groups. Model I was adjusted for admission information and severity score, including admission type, age, gender, aortic rupture, SOFA, SAPSII and GCS; Model II was adjusted for complication and operation, including sepsis, chronic pulmonary diseases, peripheral vascular diseases, hypertension, renal failure, coagulopathy, fluid and electrolyte disorders, extracorporeal circulation, bypass surgery, ventilation on first day and urine output on first day; Model III was adjusted for laboratory indicators, including bicarbonate, creatinine, blood urea nitrogen, hematocrit, hemoglobin, PTT, PT, INR, white blood cell count and platelet count; Model IV was adjusted for admission information, severity score, complication, operation and laboratory indicators above. Moreover, decision curve analysis (DCA) was drawn to show the net benefit and clinical usefulness of different models adjusted for different kinds of variables [17].

The data analysis above were all finished in R software (Version: 3.6.1).

## Results

### Enrollment

**Fig. 1:**
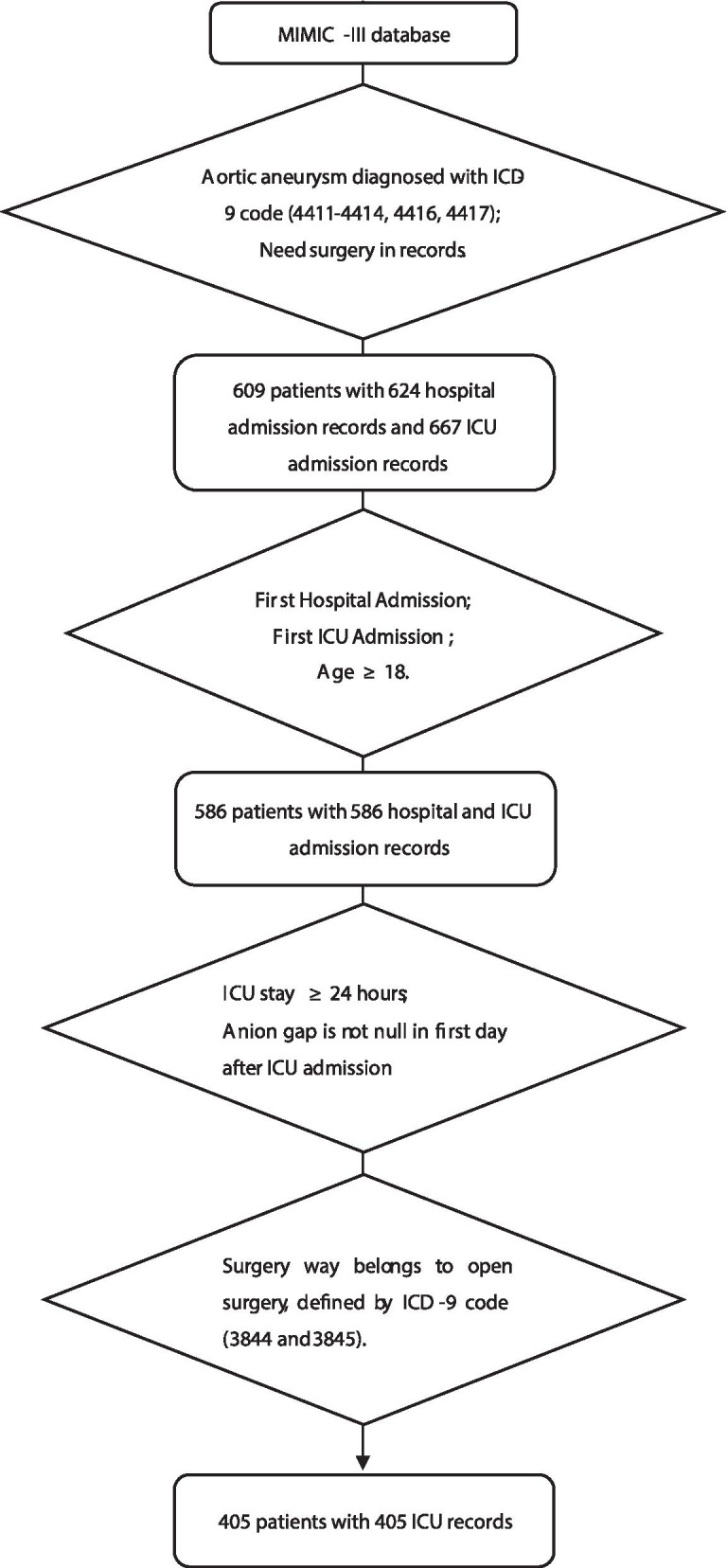
Flow chart of the study population. In-ICU AA patients with open surgery records were enrolled. Abbreviation: MIMIC-III, Medical Information Mart for Intensive Care III; ICD-9, international Classification of Diseases, 9th Revision; ICU, intensive care unit

The process of enrollment is shown in Fig. 1. Firstly, through inclusion of patients with ICD-9 code 4411–4414, 4416, 4417 (4411: thoracic aneurysm, ruptured; 4412: thoracic aneurysm without mention of rupture; 4413: abdominal aneurysm, ruptured; 4414: abdominal aneurysm without mention of rupture; 4416: thoracoabdominal aneurysm, ruptured; 4417: thoracoabdominal aneurysm, without mention of rupture) and exclusion of patients without surgery in records, 609 AA patients with 624 hospital admission records and 667 ICU admission records were obtained from MIMIC-III. Secondly, among these patients and records, first hospital admission and first ICU admission record of patients no less than 18 years old were included, while other records were excluded. Through this we obtained 586 patients with 586 hospital and ICU admission records. Thirdly, by using ICD-9 code 3844 (resection of vessel with replacement, aorta, abdominal) and 3845 (Resection of vessel with replacement, thoracic vessels), we enrolled the patients whose surgery way belongs to open surgery, and 405 patients with 405 ICU records were enrolled in the study eventually. Among them, 144 were AAA patients, 227 were TAA patients, 34 were thoracoabdominal aortic aneurysm patients.

### Baseline data

As is shown in Table 1, among these 405 patients, 377 (93.1%) survived and 28 (6.9%) died in ICU. Sensitivity analysis showed no difference between original and imputed data, which is shown in Stable 1. Comparing the ICU survival group and the death group, in general condition, admission type, death in hospital, death within 90 days, death within 365 days, LOS in hospital, age, type of AA and aortic rupture were statistically significant (*P* value < 0.05); in comorbidity, sepsis and coagulopathy were statistically significant (*P* value < 0.05); in laboratory indicators on admission, AG, bicarbonate, creatinine, blood urea nitrogen, PTT, INR, white blood cell count and platelet count were statistically significant (*P* value < 0.05); in treatment, extracorporeal circulation and urine output on first day were statistically significant (*P* value < 0.05); in severity scores, SOFA and SAPSII were statistically significant (*P* value < 0.05).

**Table 1:** Baseline data of aortic aneurysm patients in intensive care unit after open surgery

Variables		ICU-Survival (N = 377)	ICU-Death (N = 28)	*P* value
**General condition**				
	Admission type			< 0.001
	ELECTIVE	282 (74.8%)	8 (28.6%)	
	EMERGENCY	86 (22.8%)	20 (71.4%)	
	URGENT	9 (2.4%)	0 (0.0%)	
	Death in hospital	2 (0.5%)	28 (100.0%)	< 0.001
	Death within 90 days	8 (2.1%)	28 (100.0%)	< 0.001
	Death within 365 days	20 (5.3%)	28 (100.0%)	< 0.001
	LOS in hospital (days)	9.06 [6.27, 14.15]	12.00 [4.07, 19.01]	< 0.001
	LOS in intensive care unit (days)	3.08 [1.86, 6.81]	11.88 [2.65, 19.19]	0.782
	Age (years)	68.58 [59.14, 75.40]	76.80 [70.14, 80.96]	< 0.001
	Type of aortic aneurysm			< 0.001
	Abdominal aneurysm without mention of rupture	101 (26.8%)	8 (28.6%)	
	Abdominal aneurysm, ruptured	25 (6.6%)	10 (35.7%)	
	Thoracic aneurysm without mention of rupture	216 (57.3%)	5 (17.9%)	
	Thoracic aneurysm, ruptured	5 (1.3%)	1 (3.6%)	
	Thoracoabdominal aneurysm, ruptured	5 (1.3%)	2 (7.1%)	
	Thoracoabdominal aneurysm, without mention of rupture	25 (6.6%)	2 (7.1%)	
	Male	248 (65.8%)	13 (46.4%)	0.063
	Aortic rupture	35 (9.3%)	13 (46.4%)	< 0.001
**Comorbidity**				
	Sepsis	6 (1.6%)	6 (21.4%)	< 0.001
	Chronic pulmonary diseases	81 (21.5%)	7 (25.0%)	0.843
	Peripheral vascular diseases	198 (52.5%)	9 (32.1%)	0.059
	hypertension	22 (5.8%)	3 (10.7%)	0.53
	Renal failure	27 (7.2%)	4 (14.3%)	0.318
	Coagulopathy	66 (17.5%)	11 (39.3%)	0.01
	Fluid and electrolyte disorders	82 (21.8%)	7 (25.0%)	0.87
**Laboratory indicators on admission**				
	Anion gap (mEq/L)	12.00 [11.00, 14.00]	17.00 [15.75, 22.00]	< 0.001
	Bicarbonate (mEq/L)	23.00 [21.00, 25.00]	17.50 [15.75, 21.00]	< 0.001
	Creatinine (mg/dL)	1.00 [0.80, 1.20]	1.65 [1.15, 2.10]	< 0.001
	Blood urea nitrogen (mg/dL)	17.00 [13.00, 21.00]	27.00 [20.75, 34.25]	< 0.001
	Hematocrit (%)	25.00 [21.10, 29.00]	24.65 [21.00, 26.10]	0.266
	Hemoglobin (g/dL)	8.50 [7.40, 9.80]	8.35 [7.00, 8.80]	0.106
	PTT (sec)	41.45 [34.35, 51.55]	76.45 [43.55, 122.25]	< 0.001
	PT (sec)	15.80 [14.60, 17.50]	17.55 [14.75, 19.40]	0.074
	INR	1.50 [1.30, 1.70]	1.80 [1.37, 2.32]	0.026
	white blood cell count (K/μL)	12.50 [9.90, 15.70]	14.65 [10.38, 16.95]	0.048
	Platelet count (K/μL)	124.00 [89.75, 156.00]	84.50 [61.00, 120.00]	< 0.001
**Treatment**				
	Extracorporeal circulation	243 (64.5%)	7 (25.0%)	< 0.001
	Bypass surgery	14 (3.7%)	2 (7.1%)	0.692
	Ventilation in first day	356 (94.4%)	26 (92.9%)	1
	Urine output on first day (ml)	2026.00 [1393.50, 3038.75]	948.00 [344.00, 1428.00]	< 0.001
**Severity scores**				
	GCS	15.00 [14.25, 15.00]	15.00 [15.00, 15.00]	0.133
	SOFA	5.00 [4.00, 8.00]	9.00 [7.75, 11.25]	< 0.001
	SAPSII	32.00 [26.00, 42.00]	49.50 [43.75, 65.00]	< 0.001

The continuous data’s normality was tested with Shapiro–Wilk normality test. And in two independent groups compare, continuous variables with normal distribution would be represented by mean with standard deviation (SD), compared with t-test. Continuous variables with abnormal distribution would be represented by median and interquartile range, compared with Wilcoxon rank sum test. Categorical variables would be represented by frequency and percentage, compared with chi-square test. 0.2% of patients had unknown value for hemoglobin; 0.2% for platelet; 2.2% for PTT; 2.2% for PT; 2.2% for INR; 0.5% for white blood cell count; 0.4% for urine output on first day; 0.7% for GCS score. Abbreviation: LOS, length of stay; PTT, partial thromboplastin time; PT, prothrombin time; INR, international normalized ratio; GCS, Glasgow Coma Scale; SOFA, sequential organ failure assessment; SAPSII, simplified acute physiology score II

### Risk stratification

As is shown in Table 2, comparing the baseline data of 3 groups whose AG levels were respectively < 12 mEq/L, between 12–16 mEq/L, and > 16 mEq/L, all factors of general condition, including admission type, death in ICU, death in hospital, death within 90 days, death within 365 days, LOS in hospital, LOS in ICU, type of AA, age, gender, aortic rupture, were statistically significant (*P* value < 0.05); in comorbidity, sepsis, peripheral vascular diseases and coagulopathy were statistically significant (*P* value < 0.05); in laboratory indicators on admission, AG, bicarbonate, creatinine, blood urea nitrogen, PTT, prothrombin time (PT), INR and platelet count were statistically significant (*P* value < 0.05); in treatment, extracorporeal circulation used and urine output in first day were statistically significant (*P* value < 0.05); in severity scores, SOFA and SAPSII were statistically significant (*P* value < 0.05).

**Table 2:** Baseline data of studying aortic aneurysm stratified by anion gap on ICU admission

Variables		Anion gap < 12 mEq/L (N = 151)	Anion gap between 12–16 mEq/L (N = 200)	Anion gap > 16 m Eq/L (N = 54)	*P* value
**General condition**					
	Admission type				< 0.001
	ELECTIVE	121 (80.1%)	153 (76.5%)	16 (29.6%)	
	EMERGENCY	27 (17.9%)	42 (21.0%)	37 (68.5%)	
	URGENT	3 (2.0%)	5 (2.5%)	1 (1.9%)	
	Death in ICU	1 (0.7%)	8 (4.0%)	19 (35.2%)	< 0.001
	Death in hospital	2 (1.3%)	8 (4.0%)	20 (37.0%)	< 0.001
	Death within 90 days	4 (2.6%)	11 (5.5%)	21 (38.9%)	< 0.001
	Death within 365 days	6 (4.0%)	15 (7.5%)	27 (50.0%)	< 0.001
	LOS in hospital (days)	7.36 [6.12, 11.01]	9.24 [6.27, 16.87]	13.52 [9.26, 21.77]	< 0.001
	LOS in ICU (days,)	2.25 [1.33, 3.98]	3.39 [2.01, 9.18]	8.95 [3.01, 20.01]	< 0.001
	Type of aortic aneurysm				< 0.001
	Abdominal aneurysm without mention of rupture	34 (22.5%)	63 (31.5%)	12 (22.2%)	
	Abdominal aneurysm, ruptured	2 (1.3%)	12 (6.0%)	21 (38.9%)	
	Thoracic aneurysm without mention of rupture	105 (69.5%)	109 (54.5%)	7 (13.0%)	
	Thoracic aneurysm, ruptured	1 (0.7%)	1 (0.5%)	4 (7.4%)	
	Thoracoabdominal aneurysm, ruptured	2 (1.3%)	2 (1.0%)	3 (5.6%)	
	Thoracoabdominal aneurysm, without mention of rupture	7 (4.6%)	13 (6.5%)	7 (13.0%)	
	Age (years)	64.04 [56.20, 73.00]	70.35 [60.43, 76.72]	73.06 [69.39, 79.86]	< 0.001
	Male	95 (62.9%)	140 (70.0%)	26 (48.1%)	0.011
	Aortic rupture	5 (3.3%)	15 (7.5%)	28 (51.9%)	< 0.001
**Comorbidity**					
	Sepsis	2 (1.3%)	4 (2.0%)	6 (11.1%)	0.001
	Chronic pulmonary diseases	31 (20.5%)	48 (24.0%)	9 (16.7%)	0.461
	Peripheral vascular diseases	90 (59.6%)	99 (49.5%)	18 (33.3%)	0.003
	hypertension	7 (4.6%)	13 (6.5%)	5 (9.3%)	0.463
	Renal failure	7 (4.6%)	17 (8.5%)	7 (13.0%)	0.116
	Coagulopathy	19 (12.6%)	35 (17.5%)	23 (42.6%)	< 0.001
	Fluid and electrolyte disorders	29 (19.2%)	43 (21.5%)	17 (31.5%)	0.17
**Laboratory indicators on admission**					
	Anion gap (mEq/L)	10.00 [9.00, 11.00]	13.00 [12.00, 14.00]	19.00 [17.00, 22.00]	< 0.001
	Bicarbonate (mEq/L)	24.00 [22.00, 25.00]	23.00 [20.75, 24.00]	18.00 [16.00, 21.75]	< 0.001
	Creatinine (mg/dL)	0.80 [0.70, 1.10]	1.00 [0.80, 1.33]	1.50 [1.12, 2.08]	< 0.001
	Blood urea nitrogen (mg/dL)	15.00 [13.00, 18.00]	18.00 [14.00, 23.25]	24.00 [18.00, 28.00]	< 0.001
	Hematocrit (%)	24.60 [21.10, 27.00]	25.00 [21.48, 29.00]	25.60 [21.00, 27.60]	0.613
	Hemoglobin (g/dL)	8.40 [7.50, 9.65]	8.50 [7.30, 9.90]	8.50 [7.10, 9.57]	0.69
	PTT (sec)	40.70 [35.95, 50.80]	41.25 [33.45, 50.05]	62.05 [39.00, 130.43]	< 0.001
	PT (sec)	15.90 [14.85, 17.30]	15.50 [14.30, 17.12]	17.00 [14.83, 19.67]	0.007
	INR	1.50 [1.30, 1.70]	1.40 [1.30, 1.70]	1.80 [1.33, 2.30]	0.001
	white blood cell count (K/μL)	12.70 [9.90, 15.30]	12.60 [10.20, 16.00]	12.00 [9.43, 16.18]	0.817
	Platelet count (K/μL)	124.00 [97.00, 151.50]	125.00 [89.00, 157.00]	93.50 [58.25, 141.75]	0.001
**Treatment**					
	Extracorporeal circulation	120 (79.5%)	115 (57.5%)	15 (27.8%)	< 0.001
	Bypass surgery	6 (4.0%)	7 (3.5%)	3 (5.6%)	0.789
	Ventilation in first day	147 (97.4%)	186 (93.0%)	49 (90.7%)	0.104
	Urine output in first day (ml)	2205.00 [1723.50, 3217.00]	1982.50 [1268.50, 3012.50]	1006.50 [489.50, 1968.50]	< 0.001
**Severity scores**					
	GCS	15.00 [14.00, 15.00]	15.00 [14.75, 15.00]	15.00 [15.00, 15.00]	0.115
	SOFA	5.00 [3.00, 7.00]	5.00 [4.00, 8.00]	8.00 [5.25, 10.00]	< 0.001
	SAPSII	31.00 [26.00, 38.50]	32.50 [27.00, 42.00]	46.50 [40.00, 58.50]	< 0.001

The continuous data’s normality was tested with Shapiro–Wilk normality test. And in two independent groups compare, continuous variables with normal distribution would be represented by mean with standard deviation (SD), compared with t-test. Continuous variables with abnormal distribution would be represented by median and interquartile range, compared with Wilcoxon rank sum test. Categorical variables would be represented by frequency and percentage, compared with chi-square test. Abbreviation: LOS, length of stay; PTT, partial thromboplastin time; PT, prothrombin time; INR, international normalized ratio; ICU, intensive care unit; GCS, Glasgow Coma Scale; SOFA, sequential organ failure assessment; SAPSII, simplified acute physiology score II

As is shown in Table 3, comparing prediction abilities of AG and SAPSII model, in predicting in-hospital mortality, AG model had a higher AUC than SAPSII model (0.861 versus 0.833), NRI of which was 0.373 (95% confidence interval (CI) 0.009–0.738) (*P* value < 0.05); in predicting 1-year mortality, AG model had a lower AUC than SAPSII model (0.775 versus 0.780), NRI of which was 0.304 (95% CI 0.007–0.601) (*P* value < 0.05) and IDI of which was 0.095 (95% CI 0.022–0.167) (*P* value < 0.05). It indicated that AG model was more capable of predicting postoperative mortality than SAPSII model, although SAPSII model performed better in predicting 1-year mortality.

**Table 3:** Compare of risk discrimination of anion gap with simplified acute physiology score II

Outcome	Continuous AG	SAPSII					
	AUC (95% CI)	AUC (95% CI)	*P* value^a^	NRI (95% CI)	*P* value^b^	IDI (95%)	*P* value^c^
In-ICU mortality	0.882(0.818–0.946)	0.839(0.771–0.908)	0.215	0.302(-0.074–0.677)	0.115	0.118(-0.013–0.249)	0.077
In-Hospital mortality	0.861(0.785–0.937)	0.833(0.767–0.899)	0.441	0.373(0.009–0.738)	0.045	0.110(-0.011–0.232)	0.076
90-day mortality	0.809(0.722–0.895)	0.801(0.730–0.872)	0.847	0.176(-0.158–0.511)	0.302	0.082(-0.014–0.179)	0.095
1-year mortality	0.775(0.700–0.850)	0.780(0.714–0.845)	0.878	0.304(0.007–0.601)	0.045	0.095(0.022–0.167)	0.01

a. For Delong’ test of two correlated ROC curves of AG and SAPSII

b. For NRI between AG model and SAPSII model

c. For IDI between AG model and SAPSII model

Abbreviation: AG, anion gap; SAPSII, simplified acute physiology score II; ICU, intensive care unit; AUC, area under the curve; NRI, net reclassification index; IDI, integrated discrimination improvement; ROC, receiver operating characteristic

### Relationship between AG and mortality

**Fig. 2:**
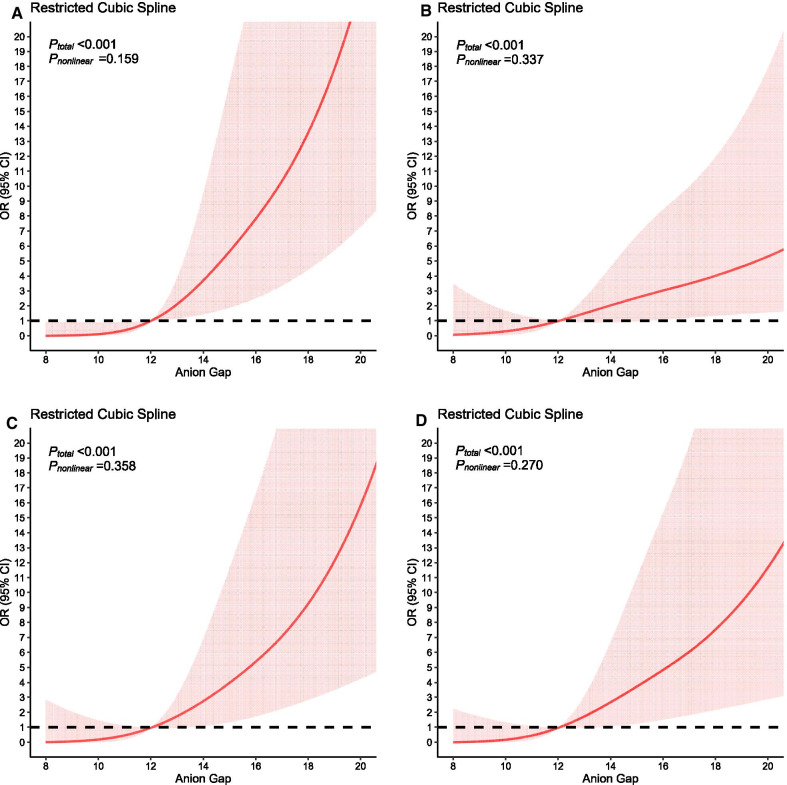
Effects of anion gap on risk of mortality shown in restricted cubic splines. **A** Non-adjusted. **B** Adjusted for admission information and severity score, including admission type, age, gender, aortic rupture, SOFA, SAPSII and GCS; **C** Adjusted for complication and operation, including sepsis, chronic pulmonary diseases, peripheral vascular diseases, hypertension, renal failure, coagulopathy, fluid and electrolyte disorders, extracorporeal circulation, bypass surgery, ventilation on first day and urine output on first day; **D** Adjusted for laboratory indicators, including bicarbonate, creatinine, blood urea nitrogen, hematocrit, hemoglobin, PTT, PT, INR, white blood cell count and platelet count. In all figures, three-nodes restricted cubic splines were conducted to flexibly model and visualize the relation of anion gap (AG) with intensive care unit (ICU) mortality. *P*_nonlinear_ > 0.05 in all models means that AG doesn’t have statistically significant nonlinear relationship with ICU mortality. Abbreviation: OR, odds ratio; PTT, partial thromboplastin time; PT, prothrombin time; INR, international normalized ratio; GCS, Glasgow Coma Scale; SOFA, sequential organ failure assessment; SAPSII, simplified acute physiology score II

As is shown in Fig. 2, RCS analysis indicated that the level of AG had a statistically significant relationship with ICU mortality (*P*_*total*_ value < 0.001). As the level of AG increased, the risk of ICU death increased at the same time. It could be seen that the growing tendency of mortality became sharper when the level of AG is above 12 mEq/L, but became gentler (still positive correction) in the RCS (Fig. 2B) which was adjusted for admission information and severity score, including admission type, age, gender, aortic rupture, SOFA, SAPSII and Glasgow coma scale (GCS).

### Subgroup analysis

**Fig. 3:**
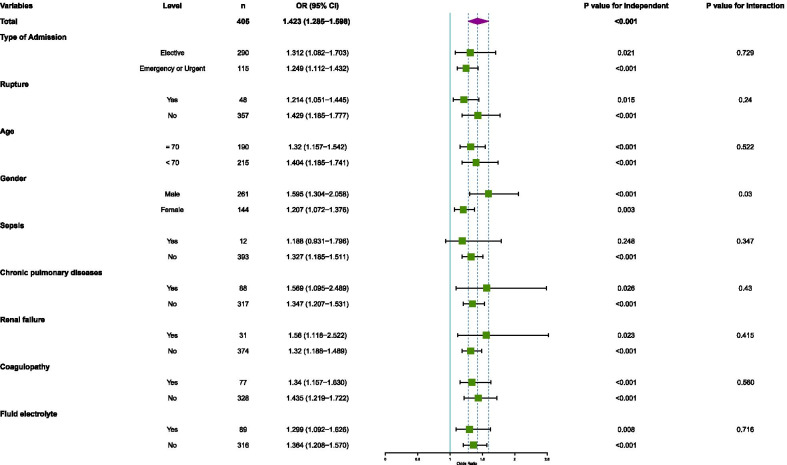
Subgroup analysis of association between serum anion gap and ICU mortality. Horizontal lines represent 95% confidence intervals. OR in each level and *P*_interaction_ were calculated after adjusting for SOFA. AG was significantly associated with in-ICU mortality in total cohort (*P* value < 0.05), all groups except sepsis identified group (*P* value = 0.248). Abbreviation: OR, odds ratio; CI, confidence intervals; SOFA, sequential organ failure assessment; AG, anion gap; ICU, intensive care unit

As is shown in Fig. 3, subgroup analysis was conducted in different groups and was shown as forest plot. AG was significantly associated with in-ICU mortality in total cohort (*P* value < 0.05), all groups except sepsis identified group (*P* value = 0.248). The results of interaction had shown that only gender had interactions with AG in predicting in-ICU mortality (*P* value < 0.05).

### Multivariate logistic regression

As is shown in Table 4, the crude model and the 4 models adjusted by other variables (Model I: adjusted for admission information and severity score; Model II: adjusted for complication and operation; Model III: adjusted for laboratory indicators; Model IV: full variables model, adjusted for admission information, severity score, complication, operation and laboratory indicators) all showed statistical significance (*P* value < 0.05).

**Table 4:** Multivariate logistic regression for effects of anion gap on intensive care unit mortality

AG	OR (95% CI)	*P* value
Crude	1.423(1.285–1.598)	< 0.001
Model I	1.199(1.063–1.380)	0.006
Model II	1.379(1.209–1.611)	< 0.001
Model III	1.316(1.142–1.546)	< 0.001
Model IV	1.286(1.053–1.651)	0.025

Crude, only includes anion gap in one model; Model I, adjusted for admission information and severity score, including admission type, age, gender, aortic rupture, SOFA, SAPSII and GCS; Model II, adjusted for complication and operation, including sepsis, chronic pulmonary diseases, peripheral vascular diseases, hypertension, renal failure, coagulopathy, fluid and electrolyte disorders, extracorporeal circulation, bypass surgery, ventilation on first day and urine output on first day; Model III, adjusted for laboratory indicators, including bicarbonate, creatinine, blood urea nitrogen, hematocrit, hemoglobin, PTT, PT, INR, white blood cell count and platelet count; Model IV, full variables model, adjusted for admission information, severity score, complication, operation and laboratory indicators above

Abbreviation: AG, anion gap; OR, odds ratio; LOS, length of stay; PTT, partial thromboplastin time; PT, prothrombin time; INR, international normalized ratio; GCS, Glasgow Coma Scale; SOFA, sequential organ failure assessment; SAPSII, simplified acute physiology score II

### Clinical benefit estimation

**Fig. 4:**
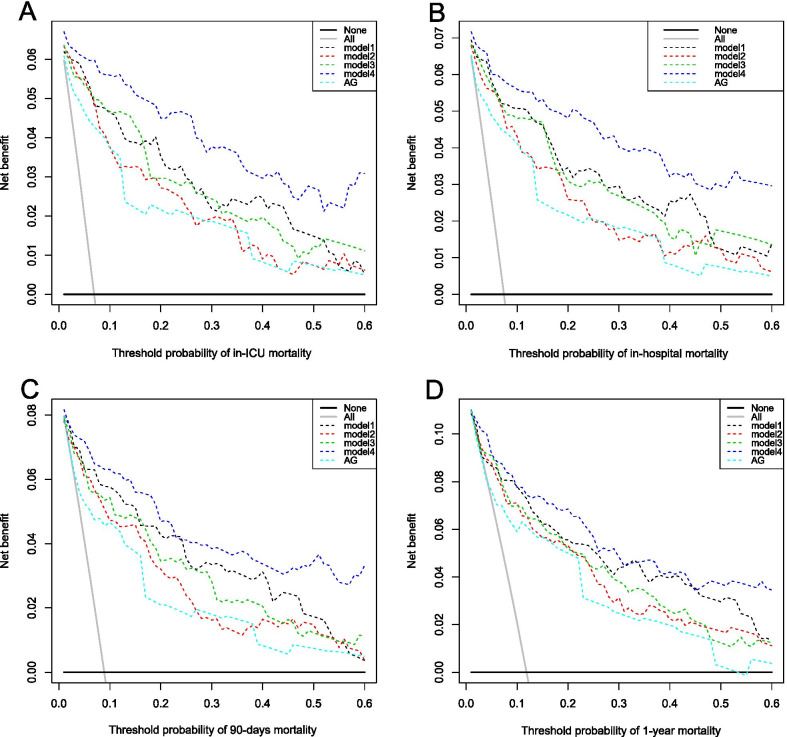
Decision curve analysis for different models with different clinical outcomes. **A** In-ICU mortality; **B** In-hospital mortality; **C** 90-days mortality; **D** 1-year mortality. Model 1 was adjusted for admission information and severity score, including admission type, age, gender, aortic rupture, SOFA, SAPSII and GCS; Model 2 was adjusted for complication and operation, including sepsis, chronic pulmonary diseases, peripheral vascular diseases, hypertension, renal failure, coagulopathy, fluid and electrolyte disorders, extracorporeal circulation, bypass surgery, ventilation on first day and urine output on first day; Model 3 was adjusted for laboratory indicators, including bicarbonate, creatinine, blood urea nitrogen, hematocrit, hemoglobin, PTT, PT, INR, white blood cell count and platelet count; Model 4 was adjusted for full variables, including admission information, severity score, complication, operation and laboratory indicators above. Compared with other models and crude AG, model 4 showed advantages in net benefit when applied to predict in-ICU mortality, in-hospital mortality, 90-day mortality and 1-year mortality of aortic aneurysm patients that had received open surgery. Abbreviation: AG, anion gap; PTT, partial thromboplastin time; PT, prothrombin time; INR, international normalized ratio; GCS, Glasgow Coma Scale; SOFA, sequential organ failure assessment; SAPSII, simplified acute physiology score II; ICU, intensive care unit

As is shown in Fig. 4, 4 DCAs showed the clinical usefulness of each model through drawing the curves which reflected the relationship between potential threshold probability of in-ICU mortality, in-hospital mortality, 90-day mortality and 1-year mortality respectively (*x* axis) and the net benefit of using the model to stratify the risk of patients (*y* axis). Through these DCAs, we could discover that in the range of risk of in-ICU mortality, in-hospital mortality, 90-day mortality and 1-year mortality, model 4 always had more net benefits compared with other models and crude AG.

## Discussion

Through multivariate analysis, we found that 1 mEq/L increase of AG improved up to 42.3% (95% CI: 28.5%-59.8%) risk of in-ICU mortality of AA patients who received OSR, which was statistically significant (*P* value < 0.001).

As we grouped patients by their admission level of AG, we found that death and LOS between those groups were all different. It proved that the subgroups divided by AG really indicated the different risk of death and hospital or ICU stay. Moreover, as we compared the risk discrimination ability of AG model and SAPSII, we found that there was no significant difference between the performance of these two models, except in hospital mortality and 1 year-mortality (AG model performed better). The AUC of AG for discrimination of survivors and non-survivor reached 0.882 (95% CI 0.818–0.946), which was higher than our previous research that studied all kinds of AA patients in ICU (AUC: 0.8513, 95% CI 0.7698–0.9328). Also, a report showed that EVAR caused smaller change of acid–base status than OSR [18]. These indicated that the prognostic prediction ability of AG might be better when dealing with patients who received OSR.

DCA and multivariate analysis both showed the good clinical usefulness and prediction ability of AG.

AG is a factor that indicates the acid–base status of patients. Traditionally, metabolic acidosis is categorized to the presence or absence of unmeasured anions, inferred by calculating AG [19]. Since it is reported that metabolic acidosis is a powerful marker of poor prognosis of critically ill patients, AG might be a prognostic factor for adverse clinical outcome in ICU patients [20].

In ruptured AAA, preoperative unmeasured AG is a prognosis factor for mortality, and elevated unmeasured AG is associated with lactic acid, ketoacids, uremia, and intoxications with nonchloride-containing acids as well as in some forms of metabolic alkalosis [21]. In our study, an evidence of this hypothesis was that different levels of AG were significantly associated with different types of AA (*P* value < 0.001), as is shown in Table 2.

Besides, several studies have indicated that aortic cross-clamp is associated with metabolic acidosis, which probably causes negative cardiovascular effects without proper intervention [18, 22]. Therefore, we inferred that an underlying preoperative acidosis, indicated by the elevated AG level on admission, might escalate when aortic cross-clamp is conducted, and eventually lead to a worse prognosis and a higher mortality when compared with the situation without preoperative acidosis. [18]

What’s more, preoperative acidosis may be a surrogate for occult systemic malperfusion ostensibly, since lactate generated from hypoperfusion generates acidosis, and the resolution of lactic acidosis contributes to survival significantly [23, 24]. In patients with acute type A aortic dissection, severe preoperative acidosis is associated with malperfusion or shock, and contributes to mortality [25, 26]. A previous report demonstrated that acidosis included by lactate, pyruvate or HCl will increase whole blood viscosity and hematocrit; therefore, although systemic flow is normal or supranormal, increase of viscosity and hematocrit may partly contribute to regional hypoperfusion [24]. Lactic acidosis as a surrogate for systemic hypoperfusion or malperfusion is becoming an increasingly recognized and useful independent indicator of disease severity and a predictor of survival in the intensive care and trauma settings [23].

Also, preoperative acidosis may have associations with coagulopathy. It is indicated that acidosis is a probable reason for coagulopathy, and bleeding caused by coagulopathy contributes to intraoperative death of ruptured AAA patients [27]. Another study reported that in disseminated intravascular coagulation patients in the cardiothoracic surgical ICU, there is a strong relationship between acid–base derangement and mortality, since the elevated blood lactate concentration and base deficit is a reflection of severe tissue hypoxia and plays a key role in survival in critically ill patients [28]. In our study, as is shown in Table 2, different levels of AG are significantly associated with different incidences of coagulopathy (*P* value < 0.001), which might be a supporting evidence of this hypothesis.

In addition to acid–base disturbance, it is reported that in geriatrics, the elevated AG level is a prognosis factor for mortality, since it is associated with hypertension, low cardiorespiratory fitness and decreased renal function [29]. Reports show that smoking, existence of other cardiovascular diseases, hypertension and dyslipidemia are risk factors of AAA, and untreated hypertension is also a risk factor for AAA rupture [1]. Besides, although smoking seems to have no significant relationship with TAA, hypertension is a risk factor for TAA, and hyperlipidemia is one of the determining factors of expansion rate of TAA, probably causing rupture [30–32]. These factors might cause negative effect on the prognosis of AA patients, and contribute to the elevated AG with the elevated mortality. The elevated incidence of cardiovascular events of AA patients is not associated to AA directly, but associated to the common risk factors and comorbidities. In the current study, although chronic pulmonary diseases, hypertension and renal failure didn’t show differences respectively between subgroups stratified by level of AG or those stratified by survival conditions, they might have a combined effect on AG and mortality, which probably explains the association between AG and mortality.

There are several limitations in this study. As this study is an observational study conducted by using an open database, we could just give a rough possible explanation of the mechanism that caused the association between AG level and mortality of AA patients in ICU after open surgery. Moreover, we did only obtain data from one database, for which we just conducted an internal validation in this study. An external validation is essential for proving the ability of our models in future studies. In addition, the net benefit of prediction models assessed by DCA was only showed through graphs, and there was not an exact numeric value that reflects the net benefit more precisely.

## Conclusions

The level of serum AG is an important prognosis factor for AA mortality in ICU after open surgery, which might promote the refinement of the existing prediction models and the establishment of new models.

## Supplementary Information

**Additional file 1.** Method - Data retrieval.

## Data Availability

The dataset supporting the conclusions of this article is freely available in the MIMIC-III database, which can be assessed on https://mimic.physionet.org. Researchers can formally request access to this free database by become a credentialed user on PhysioNet (https://physionet.org/) after completing the CITI (Collaborative Institutional Training Initiative) “Data or Specimens Only Research” course and approved by the institutional review boards of Beth Israel Deaconess Medical Center [11]. The datasets generated and/or analysed during the current study are not publicly available because we do not have access to data repositories open to the public at present, but are available from the corresponding author on reasonable request.

## Abbreviations

AA: Aortic aneurysm
AAA: Abdominal aortic aneurysm
AG: Anion gap
AUC: Area under curve
CI: Confidence interval
CITI: Collaborative Institutional Training Initiative
DCA: Decision curve analysis
DREAM: Dutch Randomized Endovascular Aneurysm Management
EVAR: Endo-vascular aortic repair
GCS: Glasgow Coma Scale
ICD-9: International Classification of Diseases 9
ICD-9-CM: International Classification of Diseases, Clinical Modification
ICU: Intensive Care Unit
IDI: Integrated discrimination improvement
INR: International normalized ratio
LOS: Length Of Stay
MIMIC-III: Medical Information Mart for Intensive Care III
NRI: Net Reclassification Index
OSR: Open surgery repair
PT: Prothrombin time
PTT: Partial thromboplastin time
RCS: Restricted cubic spline
ROC: Receiver operating characteristic
SAPSII: Simplified Acute Physiology Score II
SD: Standard deviation
SOFA: Sequential Organ Failure Assessment
TAA: Thoracic aortic aneurysm
TEVAR: Thoracic endovascular aortic repair
