# Supplementary material for: Association between anion gap and mortality of aortic aneurysm in intensive care unit after open surgery
Source: BMC Cardiovasc Disord. 2021 Sep 23;21:458. doi: 10.1186/s12872-021-02263-4 (PMC8459533; doi:10.1186/s12872-021-02263-4)
Supplement: Supplementary file 1 — Additional file 1. Method - Data retrieval. [file 12872_2021_2263_MOESM1_ESM.docx]

**STable 1** Sensitivity analysis of difference between original and imputed data.

| Variables |  | | Original data  (N=405) | Imputed data  (N=405) | *P* value |
| --- | --- | --- | --- | --- | --- |
| General condition | |  |  |  |  |
|  | | Admission type |  |  | 1 |
|  | | ELECTIVE | 290 (71.6%) | 290 (71.6%) |  |
|  | | EMERGENCY | 106 (26.2%) | 106 (26.2%) |  |
|  | | URGENT | 9 (2.2%) | 9 (2.2%) |  |
|  | | Death in ICU | 28 (6.9%) | 28 (6.9%) | 1 |
|  | | Death in hospital | 30 (7.4%) | 30 (7.4%) | 1 |
|  | | Death within 90 days | 36 (8.9%) | 36 (8.9%) | 1 |
|  | | Death within 365 days | 48 (11.9%) | 48 (11.9%) | 1 |
|  | | LOS in hospital (days) | 9.09 [6.25, 14.70] | 9.09 [6.25, 14.70] | 1 |
|  | | LOS in ICU (days) | 3.15 [1.91, 7.31] | 3.15 [1.91, 7.31] | 1 |
|  | | Type of aortic aneurysm |  |  | 1 |
|  | | Abdominal aneurysm without mention of rupture | 109 (26.9%) | 109 (26.9%) |  |
|  | | Abdominal aneurysm, ruptured | 35 (8.6%) | 35 (8.6%) |  |
|  | | Thoracic aneurysm without mention of rupture | 221 (54.6%) | 221 (54.6%) |  |
|  | | Thoracic aneurysm, ruptured | 6 (1.5%) | 6 (1.5%) |  |
|  | | Thoracoabdominal aneurysm, ruptured | 7 (1.7%) | 7 (1.7%) |  |
|  | | Thoracoabdominal Aneurysm, without mention of rupture | 27 (6.7%) | 27 (6.7%) |  |
|  | | Age (years) | 69.41 [59.58, 76.28] | 69.41 [59.58, 76.28] | 1 |
|  | | Male | 261 (64.4%) | 261 (64.4%) | 1 |
|  | | Aortic rupture | 48 (11.9%) | 48 (11.9%) | 1 |
| Comorbidity | |  |  |  |  |
|  | | Sepsis | 12 (3.0%) | 12 (3.0%) | 1 |
|  | | Chronic pulmonary diseases | 88 (21.7%) | 88 (21.7%) | 1 |
|  | | Peripheral vascular diseases | 207 (51.1%) | 207 (51.1%) | 1 |
|  | | Hypertension | 25 (6.2%) | 25 (6.2%) | 1 |
|  | | Renal failure | 31 (7.7%) | 31 (7.7%) | 1 |
|  | | Coagulopathy | 77 (19.0%) | 77 (19.0%) | 1 |
|  | | Fluid and electrolyte disorders | 89 (22.0%) | 89 (22.0%) | 1 |
| Laboratory indicators on admission | |  |  |  |  |
|  | | Anion gap (mEq/L) | 12.00 [11.00, 15.00] | 12.00 [11.00, 15.00] | 1 |
|  | | Bicarbonate (mEq/L) | 23.00 [21.00, 25.00] | 23.00 [21.00, 25.00] | 1 |
|  | | Creatinine (mg/dL) | 1.00 [0.80, 1.30] | 1.00 [0.80, 1.30] | 1 |
|  | | Blood urea nitrogen (mg/dL) | 17.00 [14.00, 22.00] | 17.00 [14.00, 22.00] | 1 |
|  | | Hematocrit (%) | 25.00 [21.00, 28.70] | 25.00 [21.00, 28.70] | 1 |
|  | | Hemoglobin (g/dL) | 8.50 [7.40, 9.80] | 8.50 [7.40, 9.80] | 0.968 |
|  | | PTT (sec) | 42.20 [34.70, 52.40] | 42.20 [34.70, 53.00] | 0.944 |
|  | | PT (sec) | 15.80 [14.60, 17.60] | 15.90 [14.60, 17.62] | 0.901 |
|  | | INR | 1.50 [1.30, 1.80] | 1.50 [1.30, 1.80] | 0.832 |
|  | | white blood cell count  (K/μL) | 12.60 [9.90, 15.80] | 12.70 [10.00, 15.80] | 0.917 |
|  | | Platelet count (K/μL) | 121.00 [89.00, 154.00] | 120.50 [88.75, 154.25] | 0.976 |
| Treatment | |  |  |  |  |
|  | | Extracorporeal circulation | 250 (61.7%) | 250 (61.7%) | 1 |
|  | | Bypass surgery | 16 (4.0%) | 16 (4.0%) | 1 |
|  | | Ventilation in first day | 382 (94.3%) | 382 (94.3%) | 1 |
|  | | Urine output in first day (ml) | 1980.00 [1300.00, 2892.00] | 1990.00 [1317.00, 2914.50] | 0.791 |
| Severity scores | |  |  |  |  |
|  | | GCS | 15.00 [15.00, 15.00] | 15.00 [15.00, 15.00] | 0.953 |
|  | | SOFA | 5.00 [4.00, 8.00] | 5.00 [4.00, 8.00] | 1 |
|  | | SAPSII | 34.00 [27.00, 43.00] | 34.00 [27.00, 43.00] | 1 |

The continuous data’s normality was tested with Shapiro-Wilk normality test. And in two independent groups compare, continuous variables with normal distribution would be represented by mean with standard deviation (SD), compared with t-test. Continuous variables with abnormal distribution would be represented by median and interquartile range, compared with Wilcoxon rank sum test. Categorical variables would be represented by frequency and percentage, compared with chi-square test. Abbreviation: LOS, length of stay; PTT, partial thromboplastin time; PT, prothrombin time; INR, international normalized ratio; GCS, Glasgow Coma Scale; SOFA, sequential organ failure assessment; SAPSII, simplified acute physiology score II.
